# A Prospective Longitude Study of Lecanemab in Early Alzheimer's Disease: 6‐month follow up

**DOI:** 10.1002/alz70861_108552

**Published:** 2025-12-23

**Authors:** Zhengluan Liao, Yanmeng Pan

**Affiliations:** ^1^ Zhejiang Provincial People's Hospital, Hangzhou, Zhejiang China

## Abstract

**Background:**

Alzheimer's disease (AD) prevalence is expected to rise dramatically due to rapidly aging populations. Alzheimer's disease pathology is triggered by the accumulation of soluble and insoluble aggregated Aβ peptides (oligomers, protofibrils, and fibrils). Lecanemab is an IgG1 monoclonal antibody, preferentially targets soluble aggregated amyloid beta (Aβ), with activity across oligomers, protofibrils, and insoluble fibrils. In the recent study, lecanemab demonstrated a consistent slowing of reduction in brain amyloid in early AD. This study aims to assess lecanemab in early AD, mild cognitive impairment due to AD and mild AD dementia.

**Method:**

Eligible patients were treated with lecanemab (10 mg/kg biweekly). The primary efficacy endpoint in the core study was change in the Alzheimer's disease assessment scale‐cognitive section(ADAS), Minimum Mental State Examination(MMSE) and Montreal Cognitive Assessment(MoCA) from baseline at 6 months. Key secondary endpoints included change from baseline at 6 months in amyloid PET Centiloids (in patients participating in the amyloid PET sub‐study).

**Result:**

A total of 50 subjects were treated with lecanemab, among whom 10 completed a 6‐month follow‐up. For the primary endpoint, there was a slowing of decline with lecanemab in cognitive at 6 months compared to baseline. For the primary endpoint, 10‐mg/kg biweekly lecanemab reduced brain amyloid in frontal cortex (−0.469 SUVr units), lateral parietal cortex (−0.361 SUVr units), lateral temporal lobe cortex (−0.374 SUVr units), medial temporal lobe cortex(−0.437 SUVr units), rear clasp back (−0.420 SUVr units), precuneus (−0.489 SUVr units), occipital cortex (−0.393 SUVr units) and cerebellar cortex (−0.174SUVr units), but it's not significant(p > .05).

**Conclusion:**

Our findings analyses demonstrated reduction in brain amyloid accompanied by a consistent reduction of clinical decline across several clinical and biomarker endpoints.

